# Improving outcomes for multi-drug-resistant tuberculosis in the Peruvian Amazon – a qualitative study exploring the experiences and perceptions of patients and healthcare professionals

**DOI:** 10.1186/s12913-019-4429-y

**Published:** 2019-08-22

**Authors:** Thomas W. McNally, Gilles de Wildt, Graciela Meza, Connie M. D. Wiskin

**Affiliations:** 1 College of Medical and Dental Sciences, University of Birmingham, Birmingham, UK; 2 Institute of Clinical Sciences, University of Birmingham, Birmingham, UK; 3 Facultad de Medicina Humana, Universidad Nacional de la Amazonia Peruana, Iquitos, Peru

**Keywords:** Multi-drug-resistant tuberculosis, Experiences, Perceptions, Healthcare professional, Patient, Relationship, Qualitative, Outcomes, Peru

## Abstract

**Background:**

Management for multi-drug-resistant tuberculosis (MDR-TB) is challenging and has poor patient outcomes. Peru has a high burden of MDR-TB. The Loreto region in the Peruvian Amazon is worst affected for reasons including high rates of poverty and poor healthcare access. Current evidence identifies factors that influence MDR-TB medication adherence, but there is limited understanding of the patient and healthcare professional (HCP) perspective, the HCP-patient relationship and other factors that influence outcomes. A qualitative investigation was conducted to explore and compare the experiences and perceptions of MDR-TB patients and their dedicated HCPs to inform future management strategies.

**Method:**

Twenty-six, semi-structured in-depth interviews were conducted with 15 MDR-TB patients and 11 HCPs who were purposively recruited from 4 of the worst affected districts of Iquitos (capital of the Loreto region). Field notes and transcripts of the two groups were analysed separately using thematic content analysis. Ethics approval was received from the Institutional Research Ethics Committee, Department of Health, Loreto, and the University of Birmingham Internal Research Ethics Committee.

**Results:**

Four key themes influencing patient outcomes emerged in each participant group: personal patient factors, external factors, clinical factors, and the HCP-patient relationship. Personal factors included high standard patient and population knowledge and education, which can facilitate engagement with treatment by encouraging belief in evidence-based medicine, dispelling belief in natural medicines, health myths and stigma. External factors included the adverse effect of the financial impact of MDR-TB on patients and their families. An open, trusting and strong HCP-patient relationship emerged as a vitally important clinical factor influencing of patient outcomes. The results also provide valuable insight into the dynamic of the relationship and ways in which a good relationship can be fostered.

**Conclusions:**

This study highlights the importance of financial support for patients, effective MDR-TB education and the role of the HCP-patient relationship. These findings add to the existing evidence base and provide insight into care improvements and policy changes that could improve outcomes if prioritised by local and national government.

**Electronic supplementary material:**

The online version of this article (10.1186/s12913-019-4429-y) contains supplementary material, which is available to authorized users.

## Background

Tuberculosis (TB) remains one of the top 10 causes of death globally, despite the availability of curative treatment [1]. Multi-drug-resistant tuberculosis (MDR-TB) accounts for a sizable proportion of TB and has rapidly become a global public health crisis [2]. In 2016 there were 10.4 million new cases of TB notified worldwide, with 490,000 classified as multi-drug-resistant [2].

MDR-TB is defined as a strain exhibiting resistance to isoniazid and rifampicin, two key first-line anti-TB drugs [3]. Therefore, patients require varying regimens of second-line therapies depending on their MDR-TB strain sensitivities. These second-line therapies, which are summarised in \ file 2, are less effective and cause more side-effects, which are more disabling. Consequently, patient outcomes for MDR-TB are worse compared to non-resistant strains. Poor treatment outcomes for MDR-TB have far reaching medical and public health implications, including higher rates of transmission in the population [4–7].

More than 95% of deaths due to TB occur in low and middle-income countries, including Peru. Peru retains the highest incidence of TB amongst Latin American countries, reaching 119 cases per 100,000 population per year [8]. Of concern, Peru remains the only South American country with a high-burden of MDR-TB, which is responsible for 10% of all TB, with up to 40% of MDR-TB patients receiving inappropriate therapies [1]. Mortality rates in some districts approach 20–55% [9, 10], compared to 4.5–17% for drug-sensitive TB [11, 12]. The incidence of MDR-TB in Loreto increased by 175% from 2013 to 2014. In 2014, 25% of patients left their treatment program and 20% of MDR-TB patients died [13]. TB prevention and treatment has improved in Peru [1, 14]. However, factors including inadequate public health strategies, limited funding and low adherence to treatment have contributed to high rates of resistance, especially in lower income regions [15–17]. Loreto, which has poor healthcare access and poverty rates reaching 63%, compared to 21.7% in Lima [18], has the highest prevalence of MDR-TB of all Peru regions, prompting local government to identify MDR-TB as a research priority in 2015 [19].

A MEDLINE literature search using the terms “MDR-TB” AND “Peru” AND “qualitative” revealed little qualitative investigation of MDR-TB management in Peru [16, 17]. The role of nurses as providers of emotional support to MDR-TB patients was explored in an interview-based study in Lima [16]. Barriers to optimal outcomes included poor adherence, quality of medical care and patient knowledge, medication side-effects, social stigma, socioeconomic factors, delayed presentation and mistrust of TB medication. Facilitators included a patient-centred approach, community and family support, a focus on psychosocial well-being through counselling for the duration of treatment and patient feelings of autonomy and control. *Horter* et al. added that hope of a cure, high-quality knowledge, patients’ belief in treatments and being involved in the decision-making process improved adherence [20]*.* Conversely, a hierarchical healthcare professional-patient relationship, where patients felt unable to share concerns through fear of judgment, adversely affected adherence [21]. More aggressive treatment regimens with more drugs at higher doses have been shown to reduce all-cause mortality for MDR-TB patients [22]. Good patient education has improved outcomes by helping to allay mistaken health beliefs and myths [23, 24] and promote patient belief in their ability to be cured [5, 25–27]. In a systematic review investigating adherence to TB treatment, *Munro* et al. stressed the importance of personal factors, for example knowledge and motivation, as well as structural factors beyond the control of the patient including poverty, accessibility of care and the organisation of the local, regional and national government MDR-TB strategies [23]. Patients with comorbidities, for example HIV or diabetes mellitus, also demonstrate worse outcomes with MDR-TB [28, 29]. The financial impact of MDR-TB on patients is also an important barrier to successful treatment, especially in Peru [16, 25, 30–32]. MDR-TB patients are subject to extreme financial and employment hardship. A 2014 study investigating the financial impact of MDR-TB on patients and their families revealed that the majority of patients have to leave their job, move house and sell their possessions in order to afford accessing their treatment [25].

While current evidence identifies factors that influence adherence to treatment, there is limited understanding of the patient perspective on MDR-TB treatment and other factors that influence patient outcomes. The perceptions of patients have also not been compared to those of HCPs, which could provide a valuable insight into the dynamic of the HCP-patient relationship.

Research to date into MDR-TB in Peru is limited to Lima, which has a differing socio-demographic profile, dissimilar cultural attitudes and increased healthcare funding, and may not be generalisable to other regions [19]. In order to halt the MDR-TB epidemic in Loreto, research into patient and HCP experiences and perceptions in the region is needed. This study aims to explore the experiences and perceptions of MDR-TB patients and HCPs in Loreto in order to identify barriers and facilitators to achieving optimal outcomes and to help inform future management strategies.

## Methods

A qualitative study was undertaken to improve understanding of barriers and facilitators to achieving optimal outcomes by investigating the perceptions, experiences and beliefs of patients and their dedicated HCPs regarding MDR-TB [33]. Semi-structured interviews were conducted with MDR-TB patients and HCPs between January and March 2018.

### Setting

The study location was Iquitos, capital of the Loreto region of the Peruvian Amazon. It is the largest urban area in Loreto with an estimated 2016 population of 437,000 [34]. Participants were recruited from the San Juan, Moronacocha, Belen and Nanay districts, areas with high rates of MDR-TB.

### Population and sampling criteria

Interviews were conducted with patients (past or present) and HCPs attending or working at primary healthcare centres in the 4 districts above.

Purposive sampling was used to achieve maximum variation in response and explore emerging themes following interim analyses [35], while providing a balance of HCPs and patients for comparative purposes. Patient participants were recruited to facilitate variation in perspectives across education status, occupation, age, gender and socioeconomic status. Patients were excluded if younger than 18 years, unable to speak Spanish or English fluently, lacking capacity to give informed consent or suffering from another illness which could affect their ability to conduct a meaningful interview or cause harm or stress. HCPs were purposively recruited to include different clinics and professions. Participants were recruited in both groups until data saturation was reached [36].

### Recruitment

Patient and HCP participants were approached by the principal investigator (TM) and given written information about the study. Potential participants who were prepared to proceed gave informed written consent.

Interviews were arranged at a time and location convenient for the participant. For patients, these were undertaken either in a private room at their health centre or in their home. For staff, interviews were in their place of work.

### Data collection

Semi-structured, face-to-face interviews were carried out to allow participants to express their views without being influenced or inhibited by others [37]. Interviews examined participants’ perceptions, beliefs and experiences of MDR-TB and their views on factors that influence patient outcomes both positively and negatively. The exploratory nature of the research question called for an open approach to questioning since the aim was generation of new theory.

All interviews were carried out in Spanish, with the help of a translator. TM asked questions in English, the translator translated them into Spanish for the participant and then translated the participants’ responses into English for TM. Prior to the first interview, the translator signed a confidentiality agreement. The information sheet, consent form, demographic questionnaire and topic guide (Additional file 1) were translated into Spanish before data collection began and reviewed by the translator to ensure clarity and comprehensibility.

A topic guide was used for all interviews, which ensured that all important areas were discussed while allowing the participant to lead the conversation to subjects important to them [38]. The topic guide covered factors already shown to influence MDR-TB patient outcomes, while encouraging the participants to discuss ideas not represented in the literature. The topic guide was piloted on Day 1 and modified to improve clarity and acceptability of the questions and prevent interview misdirection [39, 40]. The quality of the translation was checked using a transcription of the pilot interview and the audio recording.

The average interview duration was 32 min (range 20–50 min). This limited the demand on participants while allowing them to explore factors in as much detail as they wished. All interviews were audio-recorded after consent. Immediately after each interview TM recorded field notes, observations and reflections to increase the accountability and trustworthiness of the findings [40]. TM interacted only as a researcher and not as a clinician in order to maintain independence and to reduce study bias.

### Data analysis

TM transcribed the English components of the interview audio-recordings and field notes within 24 h of the interview. Spanish parts of the conversation were transcribed by the translator. The accuracy of the translation was checked for 50% of transcriptions to ensure data validity and discrepancies were rectified by discussion.

Thematic content analysis was most appropriate for this study design to provide a rich understanding of perceptions, beliefs and experiences [41]. Themes were developed and refined using the 6-step approach proposed by Braun and Clarke [41].

Data analysis occurred alongside data collection in an iterative process to inform sampling for future interviews and to assess data saturation. Constant comparison of codes and theory from interim analyses within and between interviews helped to test and generate new theory [41]. Analytical memos were kept to track the development of theory and to ensure transparency. Analytical triangulation was carried out with CI who independently analysed transcripts. Discrepancies were resolved via discussion. Transcripts were read and re-read to actively search for discrepancies within the data, contradictory or unexpected findings and to ensure that findings were a true reflection of the data and participant voice [33].

### Ethical considerations

The study was approved by the Institutional Research Ethics Committee, Department of Health, Loreto on the 8th January 2018 and The University of Birmingham Internal Research Ethics Committee on the 12th January 2018. Data were stored according to University of Birmingham data protection policy [42].

## Results

Twenty patients with MDR-TB were identified from local registers for recruitment. Five could not be traced. No participants were excluded on the basis of language. Of 15 patients interviewed 14 had confirmed MDR-TB of whom 12 were receiving active treatment and 2 had been successfully treated. Data from one patient interview (participant 023) was excluded from the final analysis when a review of the records revealed they had mono-resistant TB. The age range of patients was 19 to 71 years, with 10 males and 4 females. Table 1 summarises the demographic data of patient participants.

**Table 1:** Summary of demographic characteristics of patient participants

Group (participant number)	Age	Address	Education
Patient – 1 (002)	25–34	Belen	University
Patient – 2 (003)	18–24	San Juan	University
Patient – 3 (004)	65–74	San Juan	Primary
Patient – 4 (006)	55–64	Moronacocha	University
Patient – 5 (007)	55–64	Belen	Secondary
Patient – 6 (009)	18–24	San Juan	Secondary
Patient – 7 (010)	35–44	Belen	Secondary
Patient – 8 (013)	25–34	San Juan	Secondary
Patient – 9 (015)	18–24	Nanay	Secondary
Patient – 10 (018)	45–54	Nanay	Secondary
Patient – 11 (019)	45–54	Moronacocha	Secondary
Patient – 12 (022)	55–64	Moronacocha	Secondary
Patient – 13 (025)	45–54	Nanay	Primary
Patient – 14 (026)	55–64	Moronacocha	Secondary

Eleven HCPs were recruited including 8 dedicated TB nurses and 3 TB physicians. Table 2 summarises the demographic data of HCP participants. Twenty-six interviews were conducted in Spanish by TM and the translator.

**Table 2:** Summary of demographic characteristics of HCP participants

Group (participant number)	Age	Address	Education
HCP – 1 (001)	25–34	Iquitos	University
HCP – 2 (005)	35–44	Belen	Other (Nurse apprenticeship)
HCP – 3 (008)	35–44	San Juan	University
HCP – 4 (011)	25–34	Iquitos	University
HCP – 5 (012)	35–44	San Juan	University
HCP – 6 (014)	25–34	Iquitos	University
HCP – 7 (016)	45–54	Iquitos	Other – (Nurse apprenticeship)
HCP – 8 (017)	45–54	San Juan Bautista	University
HCP – 9 (020)	55–64	Iquitos	University
HCP – 10 (021)	25–34	San Juan	University
HCP – 11 (024)	35–44	Punchana	Other (Nurse apprenticeship)

Separate analyses of the two groups generated 4 identical key themes: 1) personal patient factors; 2) external factors; 3) clinical factors; and 4) HCP-patient relationship. Patient and HCP themes and subthemes are summarised in Tables 3 and 4 respectively. Since sampling was purposive, the numbers of respondents expressing certain views indicate whether a theme was rarely or commonly expressed, rather than indicating importance. Where appropriate, quotes from both participant groups have been paired to illustrate similarity or difference in perception between groups.

**Table 3:** Themes and subthemes from patient interviews and patients’ views of their influence on MDR-TB outcomes. (Brackets indicate the number of patients highlighting a subtheme in their interview, **bold text** indicates alignment of opinion between patients and HCPs)

Theme	Facilitators to achieving optimal outcomes (frequency out of 14)	Barriers to achieving optimal outcomes (frequency out of 14)
Personal Factors	**− High standard patient knowledge, understanding of resistance and of the importance of adherence to the end of the treatment schedule** (10) **−** Education from a number of sources including the HCP, peers and internet (8) **−** Perception of MDR-TB as a dangerous and contagious illness (11) **− Belief in pharmaceutical medications** (9) **− Positive perception of the future and hope in a cure** (10) **− Positive patient attitude and a strong desire to be cured** (7) **− Psychological resilience** (11)	**− Poor quality or limited knowledge** (10) **− Contradictory advice/education from peers/family/HCPs** (10) **− Belief in natural medicines more than/as much as pharmaceutical medications** (7) **− Disbelief/distrust of pharmaceutical medications** (11) **−** Myths and misinformation (10) **− Negative patient attitude** (6) **− Change in patient identity, not feeling “normal”** (9) **− Experience/fear of stigma or discrimination** (11) **−** Psychological impact of MDR-TB (12) **− Isolation** (11) **−** Other comorbidities (4)
External Factors	**− Family support (emotional/psychological/nutritional/financial)** (11) **−** Family willing to make sacrifices to help patient (8) **− Education of family to empower them to help patient** (9) **−** Religion as a source of psychological support (6) **− Holistic care provided by HCP team** (10) **− Individualised care, patient feeling involved in treatment planning** (9) **− Pragmatic approach to providing high standard care in a resource poor setting** (5)	**− Socioeconomic impact of illness and treatment** (14) **− Poor nutrition** (10) **− Resource poor health system** (7) **− Lack of political will to tackle MDR-TB/TB on a regional/country level** (6) **− Impact of illness on family (socially/psychologically/financially)** (12) **−** Disorganised care (7) **− Difficulty accessing treatment** (8)
Clinical Factors	**−** Feeling mentally and physically prepared for the treatment (9) **− Strategies to reduce nausea due to treatment** (10)	**−** Diagnosis at late stage of disease (7) **− Side-effects of the treatment** (14) **− Long duration of treatment** (11)
HCP-patient Relationship (13)	**− Good, open and trusting HCP-patient relationship** (13) **− Patient feeling comfortable sharing worries and concerns with HCPs without fearing judgment** (12) **− Patient feeling valued** (11) **− Good communication with HCP** (12)	**− Patient fear/experience of stigmatisation/discrimination from HCPs** (5)

**Table 4:** HCP themes and subthemes and their influence on MDR-TB outcomes. (Brackets indicate the number of HCPs highlighting a subtheme in their interview, **bold text** indicates alignment of opinion between HCPs and patients)

Theme	Facilitators to achieving optimal outcomes (frequency out of 11)	Barriers to achieving optimal outcomes (frequency out of 11)
Personal Factors	**− High standard patient knowledge and understanding** (10) **− Appreciation of the importance of adherence until the end of the treatment schedule** (8) **−** Knowledge of TB and MDR-TB prior to diagnosis (5) **−** Targeted education of at-risk groups (4) **− Belief in the efficacy of pharmaceutical medications** (8) - **Positive patient attitude, self-responsibility** **and desire to be cured** (9) **− Psychological resilience** (6) **−** Information seeking behaviour (8)	**− Poor level of understanding of resistance and the importance of adherence** (9) **−** Education in non-simple language and not confirming patient understanding (7) **− Contradictory advice from family or peers** (8) **− Lack of belief in health services and/or trust in alternative medicines** (9) **− Patient experience of or fear of stigma, discrimination or isolation** (9) **− Change in patient identity** (7) **−** Doubt or denial of diagnosis (6) **− Psychological impact of MDR-TB** (7)
External Factors	**−** Effective teamwork within the HCP team/health Centre (7) **− Ingenuity and pragmatism in working in a resource poor setting** (4) **− High standard family support (emotional, psychological, financial and nutritional)** (10) **− Holistic approach to care** (9) **− Home visits** (7) **− Individualised/personalised patient treatment plan** (10)	**− Resource poor health system** (9) **− Lack of political will to improve TB care on a regional/national government level** (4) **− Difficulty accessing treatment due to the financial impact of transport** (9) **− Socioeconomic impact of having MDR-TB** (10) **−** Patient prioritizing other responsibilities over health (7) **− Poor nutrition** (10) **− Impact of illness on patients’ families** (6)
Clinical Factors	**−** Observed treatment (7) **− Strategies to reduce side-effects** (8)	**− Long duration of treatment schedule** (8) **− Side-effects of the treatment** (11) **−** Disabling illness (9)
HCP factors	**− A good, open and trusting HCP-patient relationship** (11) **− Good communication** (10) **− The patient feeling valued and comfortable sharing concerns and worries with the HCP** (9) **−** Reducing transmission within the population (7) **−** Following safety protocols to prevent HCP transmission. (8)	**−** Poor communication (7) **− Patient experiencing/fearing stigmatisation from HCPs** (5)

## Personal patient factors affecting outcomes

This theme explores patient contributed factors that can influence outcomes of MDR-TB treatment including patient knowledge, beliefs about or attitude towards MDR-TB and its treatment.

### Poor patient knowledge and information

Ten patients exhibited limited knowledge and understanding of MDR-TB with a proportion of patients unable to offer even a simple description of the disease:

***Researcher*** *: What do you understand about tuberculosis as a disease?*

*Well, I can’t really tell you anything about it. (025 - patient)*

*I don’t know how to explain what causes it because I don’t know how I got it. 004 (patient).*

Resistance is a difficult concept and only 4 patients were able to explain the term correctly. Most patients understood MDR-TB as “*stronge*r” or “*harder to cure*”. This finding was echoed in interviews with HCPs who described a limited level of understanding of MDR-TB in the general population:

*[The] nurse gives the patients who come to the health centre enough information about the symptoms … If they don’t come, they don’t develop a good understanding. (017 - HCP)*

Some patients blamed their poor level of knowledge on inadequate education from HCPs:

*Some of them explain to you, but some of them don’t. I don’t know very much about this illness. (013 - patient)*

Most patients showing poor knowledge saw education as the responsibility of HCPs and did not actively seek out information about their illness. Patients who exhibited a good level of understanding saw education as their responsibility and actively sought information from a number of sources, particularly the internet:

*When I came here, and the doctors gave me the diagnosis, they didn’t tell me about the illness and what I could do … And in my case, I searched the internet. That’s why I know what this illness is about. (003 - patient)*

### Good patient knowledge and education

Despite gaps in some areas of knowledge, 10 patients mentioned the importance of adherence to the end of the treatment schedule. Furthermore, HCPs indicated that often patients suspected they had TB prior to their diagnosis, indicating a prior knowledge of symptoms and disease progression:

*When you tell patients that they have tuberculosis, usually they already know about it … They usually say, “I guessed I had that, I thought it was tuberculosis”. (001 - HCP)*

The importance of high standard education and patient understanding was stressed by 10 HCPs and 6 considered it among the most important factors for achieving optimal outcomes. HCPs described quality education as establishing knowledge of TB and MDR-TB transmission, the necessity of adherence and consequences of poor adherence, side-effects and of the efficacy of pharmaceutical medications. Through developing a good understanding, patients are more accepting of side-effects and are more optimistic about their outcome:

*It is very important because depending on the education we give to all patients and their families; they will successfully follow the treatment. (008 - HCP)*

*We try to explain to them that if they have tuberculosis they can only be cured if they follow the treatment. (005 - HCP)*

HCPs also stressed the importance of the method of delivery and source of the information. Four HCPs described using simple language to ensure patients could grasp the difficult concepts:

*I try to explain it in simple language, so they can understand what they have … Sometimes they are not able to understand clearly because we use … some technical words related to medicine. (014 - HCP)*

HCPs emphasised the importance of educating the population as a whole, while patients spoke mainly of education of individuals and their families. Both groups, however, agreed that education of families was important to enable them to support the patient more effectively:

*We try to explain the illness and to educate the family, too. We explain things related to this illness, so the patient can feel in a good environment with their family and … in their home. (011 - HCP)*

*Yes, it’s very very important [education of the population]. We need to go to schools and educate the whole population. (014 - HCP)*

Schools were frequently mentioned as good locations to educate the population about MDR-TB. In addition, both HCPs and patients mentioned the internet as a good source of information:

*When I found out I had this illness, I searched the internet for information about it … At the beginning I thought this illness was like dengue or malaria, but … now I am well informed. (003 - patient)*

### Patient health beliefs and alternative medicine

An initial distrust of medical advice and pharmaceutical medicines was evident in 11 patient interviews, while HCPs admitted difficulty explaining the efficacy of pharmaceutical over natural medicines. This initial distrust stemmed from strong cultural beliefs in natural medicines and shamans, who are natural healers in the Amazon region:

*The belief of the whole population here is that first, if they have an illness, they must go to see a shaman and then after that they come to the health centre. (017 - HCP (doctor))*

*Well, we are in Peru, aren’t we? … Here we have a huge variety of natural medicines … I learnt about natural medicines thanks to my grandmother… When they didn’t have the opportunity to go to a health centre or a hospital, they used to cure illnesses using natural medicines. (009 - patient)*

Some patients justified their belief in natural medicines by proposing that previous generations survived conditions such as tuberculosis using just natural medicines, so they should be able to as well. These cultural beliefs are compounded by contradictory advice from friends, family and patients’ communities:

*People tell me that, “you are going to die taking that medication. You are going to be blind and you are going to die.” (007 - patient)*

While it is true that some MDR-TB medications, such as ethambutol, can cause optic neuropathy and blindness, patients must tolerate these side-effects in order to rid their body of MDR-TB. It was evident in some interviews that patients and families incorrectly associated medication side-effects with the patient’s overall condition worsening:

*They [my family] tell me, for example … that I shouldn’t take the pills from the health centre here. They tell me that it’s better to take natural medicines. (010 - patient)*

Four patients revealed a belief that natural medicines were “*natural*” and therefore safer and more effective than pharmaceutical medicines***.*** Other patients argued that the active ingredients in many pharmaceutical medications are found in many natural medicines used in the Amazon, so natural medicines are a more “*natural*” way of achieving the same effect. Six patients admitted stopping the treatment from the health centre at some point during their schedule to seek a cure in natural medicines:

*The 15 days I didn’t come to the clinic I was taking natural medicines instead. (007 - patient)*

Alternatively, patients take natural medicines alongside pharmaceutical medicines either in the hope that the side-effects of the pharmaceutical medicines are made more manageable, or that they will be cured faster due to the combined effect of the two types of medicine:

*When I started this treatment here at the clinic… I had stomach ache and pain and other side-effects. The shaman told me that I needed to take natural medicines … So now I am taking both medications. 002 - patient)*

*I am following the treatment but at the same time I am taking these natural medicines. I think that this way I will be cured faster. (013 - patient)*

HCPs agreed with this:

*Sometimes they tell us that they prefer to take natural medicines because of the side-effects of the medications they take that we prescribe … That’s why I think patients often combine both … to help to deal with the side-effects. (001 - HCP)*

However, natural medicines appeared to be a cause for concern for many HCPs who expressed worry about their dangers:

*It is dangerous because they don’t know how strong it could be or dangerous it could be taking those roots or plants. They could get gastritis or any other illness because of the effects of the traditional medicines. (008 - HCP)*

As a result of the strength of patients’ belief in natural medicines, HCPs explained that it is more effective to offer a compromise and to tell patients that they can take natural medicines, but only once they have finished their course of pharmaceutical medicines:

*We respect their beliefs about the traditional medications, but we explain to the patients that if they want to take traditional medications then they can do it, but after finishing all of the treatment from us at the health centre. (008 - HCP)*

Despite the initial distrust, patients who were nearing the end of their treatment schedule believed in the efficacy of pharmaceutical medicines. Once patients could notice an improvement in their condition, they found it easier to believe in them. Conversely, if patients stop their treatment from the health centre to take only natural medicines, they notice a deterioration in their condition and go back to pharmaceutical medicines:

*I have a message for all the patients who have MDR-TB. They shouldn’t take the natural medicine because it is a lie … and I almost died. (007 - patient)*

*travelled to a village to live there. There I didn’t take the medication from the clinic, instead I took natural treatment based on vegetables and healing plants, but then I got worse and I came back to the city to follow the treatment schedule from the health centre. (004 - HCP)*

### Psychological impact, character and attitudes

Twelve patients described a significant psychological impact due to MDR-TB that begins with diagnosis and continues for life, which was reinforced by HCPs. Many patients explained difficulty coming to terms with not being a “*normal*” person and being labelled as “*ill*”:

*I am not like a normal person … Everything has changed. (015 - patient)*

The change in identity is compounded by the effect that MDR-TB and its treatment have on patients’ role both in their family and society following their diagnosis due to their inability to work or study and the fear of passing the illness on to someone else:

*I cannot hold her [my daughter]. I want to be with her as a mother. I want to be able to prepare meals for her and she wants to be with me. It’s very very hard. (013- (patient)*

Eleven patients also spoke of feeling isolated in their own homes, which has a significant psychological impact:

*When you have this illness you feel frustrated, like you are in an enclosed box. (009 - patient)*

Stigma and discrimination were also reported by 11 patients and 9 HCPs. This, combined with feelings of isolation, caused patients to lose their sense of self-worth:

*Yes, it [stigma] affects patients too much. They feel diminished in society. (016 - HCP)*

The importance of a positive patient attitude was highlighted by 7 patients and 9 HCPs. Both groups agreed that belief in oneself and a strong desire to be cured helps a patient overcome the challenges of the MDR-TB treatment schedule:

*I think that it only starts working once you want to get better. (002 - patient)*

HCPs spoke at length about the importance of instilling a positive attitude for keeping patients motivated to stay on the treatment regimen:

*The patients who stay on the treatment are the ones who have a positive outlook. Patients who have a negative attitude are the ones who stop taking the treatment. (001 - HCP)*

Four patients described their source of motivation as ensuring the safety and future security of their family:

*You think about your family when you have this illness because if you die who is going to support the family?... You need to finish the treatment. (018 - patient)*

Both patients and HCPs mentioned difficulty managing MDR-TB alongside other comorbidities such as diabetes, HIV and drug or alcohol dependence. Problems arise from difficulty with nutrition, polypharmacy, worse general health and poor engagement with the treatment program:

*Because of my diabetes I need to be careful when I feed myself. I cannot drink milk or fruit juice or eat any other fruit like a normal patient. (007 - patient)*

*We had some patients who were addicted to drugs. All 8 of those patients left the treatment. I would say that they did not feel engaged enough to continue with the treatment. That’s why they left. (012 - HCP)*

In Iquitos, there are high rates of alcohol and illicit drug abuse, especially in areas with high rates of poverty. HCPs highlighted alcohol abuse as an important barrier to treatment compliance, as well as cocaine, cannabinoid and opiate abuse.

### External factors affecting outcomes

External factors describe elements that are out of the control of the patient that influence outcomes, including socioeconomic, structural and support factors.

### Socioeconomic factors

The socioeconomic impact of MDR-TB was mentioned by all 14 patients and 11 HCPs. Both groups described how MDR-TB affects patients financially by compromising their ability to work and the added costs of accessing treatment. It became evident that both HCPs and patients agreed that the financial strains of MDR-TB affect poorer patients most, making it harder for them to complete their treatment compared to more wealthy patients. Both the illness itself and the side-effects of the treatment are disabling and frequently leave patients too weak to leave their beds. Rest is also an important part of MDR-TB treatment, so patients are encouraged not to work during the initial intensive phase of their treatment by HCPs during the early stages of their treatment:

*I don’t work like I did before. I can’t carry on working every day. I can’t earn enough money like I used to when I worked every day. (015 - patient)*

*Most of the people who live here sell things at the market. So … the first two months we tell them that they need to rest and cannot work. (008 - HCP)*

As well as the financial implications of being unable to work, patients must also pay for transport to and from the clinic every day for their medication:

*Well, when I didn’t have this illness, I had money … Years ago I had a boat, other things and possessions. Then, because of the illness, I had to sell it all to get money to pay for the treatment, pay for the transport and so on … ” (004 - patient)*

Seven patients described being unable to take the recommended rest during their treatment because they needed to work to provide for their family:

*The doctor told me to take a rest for around 30 days. I couldn’t because of my family. I have to work every day to support them. That’s why I think my treatment took 28 months because I had to work, which made it difficult to follow the treatment. Because if I don’t work, who is going to support my family? (018 - patient)*

Ten patients described not having enough money to pay for a healthy diet to ensure good nutrition, or even to buy 3 meals a day:

*I didn’t have enough money to eat and sometimes I would eat just once, at night. (018 - patient)*

*The nurse told me that I should eat at least 5 times a day. I laughed. I asked how could I possibly do that if I don’t have the money to do that? I wasn’t able to do it because the financial aspect is so important for anyone’s treatment. (026 - patient)*

Both patients and HCPs agreed that the limited nutritional support from the health centre was helpful. However, both groups also stressed that it was insufficient, and that financial support is necessary:

*There are cases when the patients need to work and sometimes, they can only afford to eat once a day, which leads to malnutrition … They don’t have the support of the government... That’s why I say this system is not working here in my country. (014 - HCP)*

*If you don’t receive financial support from anyone, it’s not enough to just receive rice and other products from the health centre during your treatment. (026 - patient)*

### Structural factors

Structural factors encompass organisational, regional and national level issues affecting the care that patients receive. Access to treatment was highlighted as a barrier to optimal outcomes not only due to financial reasons, but also logistical problems:

*I also live by the road about 10km from here. I am so far from the health centre, but I need to come here to take my medicines so it’s very very difficult for me. (013 - patient)*

*We have this problem with flooding in this neighbourhood, so we can’t visit all the patients or even a patient can’t come to the health centre. (016 - HCP)*

Working in a resource poor health system, as in Iquitos, presents a number of challenges that can affect outcomes. Both HCPs and patients described the lack of specialised personnel in the region leading to long waiting times for appointments:

*There is just one doctor who specialises in this illness, who works at all the clinics and with all the patients. It’s not easy to get an appointment with him. (002 - HCP)*

Furthermore, the environment for consultations and the general health centre facilities were also raised as concerns for reasons of privacy and confidentiality and the storage and maintenance of medication:

*I think it would be better if each of us had our own room, so we could see patients in private, so we could interact with them and they can feel more comfortable with sharing sensitive information. (001 - HCP)*

*Sometimes here at the clinic we don’t have clean water. Also, my fridge is not working so sometimes I can’t store certain medications. (014 - HCP)*

Both HCPs and patients also spoke of the risk of nosocomial infection:

*The risk is almost constant. It is risky because you don’t know. Sometimes patients come in without wearing a mask because they are not diagnosed with this illness yet. (017 - HCP)*

*The infrastructure could be improved because the doctors work in a closed room so it’s easy for the doctors and nurses to catch the illness. The room is very small and poorly ventilated so it’s not great for the doctors’ and nurses’ health. (002 - patient)*

Seven patients also mentioned disorganisation of the local health system as adversely affecting their care:

*Well, at the beginning I would say that it [my care] was not well organised … That’s why I didn’t take my medication and that’s why I think I have the same illness again for a second time. (003 - patient)*

Both national and regional politics were cited as barriers to achieving optimal outcomes. Patients and HCPs stressed that the government must do more to combat the MDR-TB problem in Peru:

*I think that it is down to the government. The government should invest more money in order to improve the healthcare system here in Peru. (009 - patient)*

*Politics should focus first on the health of the population. For example, here health should be in first place, but in Peru I think it is in third place. (017 - HCP)*

### Support

Family support was mentioned in 10 patient and 10 HCP interviews as being important. Patients and HCPs highlighted that a diagnosis of MDR-TB affected the patients’ families in many ways including the risk of transmission, stigma and discrimination by association and also the necessity of making sacrifices to provide support:

*It is common for families to have to make sacrifices to support family members with tuberculosis. (001 - HCP)*

A positive reaction by the family and provision of support was recognised as being important by both groups:

*Definitely. It [family support] is very important because sometimes when the patient feels sad and terrible it’s obvious that they need family support. Sometimes that can be the reason they don’t finish the treatment if they don’t have family support... The family needs to be with the patient during their whole treatment … to support the patient psychologically or financially. (021 - HCP)*

*The only person who stayed with me since I got this illness is my wife. This is so important because there are many friends who leave you and don’t want to be friends any more. But, it is worse when a relative discriminates you. That makes you feel so bad. (002 - patient)*

Family support was described in many forms including: emotional, psychological, financial and nutritional:

*First of all, my family helped me psychologically. My husband, my sisters, my daughters … All my family helped me. They said, “continue with your treatment. Don’t leave your treatment.” (007 - patient)*

Six patients highlighted religion as providing them with psychological support:

*When I feel alone, I only trust one person, and that is God. When I wake up, I think of God. He is the only one I trust... Thanks to God I am here. (009 - patient)*

Both groups also highlighted the importance of the quality and nature of the care and support provided by the HCP team. Ten patients and 9 HCPs stressed that care provided by the HCP team must be holistic, incorporating a multi-disciplinary team that supports the patient emotionally and psychologically as well as medically:

*When the patient is diagnosed with this illness, they receive a complete treatment not just in this area with the nurses, but they also receive support from other doctors in other specialties. All the staff support the patients here. (011 - HCP)*

*We try to support our patients. We give them some advice, but it’s not enough. We have here a specialist, a psychologist; she is here all the time trying to talk to the patients … Most of the patients with this illness feel very bad, terrible. (020 - HCP)*

Care beyond the health centre and home visits were also raised as key aspects of the care package:

*If we are worried about the patient’s health, we visit them … and we look for what is happening with that patient. Maybe he/she cannot travel to the clinic, for example. We need to know why that patient is not coming to the health centre. Maybe he/she feels too weak to come to the health centre and that’s why we visit them. It is our work. (005 - HCP)*

Nine patients also spoke of a pragmatic and individualised treatment plan as being particularly helpful and they valued involvement in its planning. For example, when a patient’s job means they cannot access the clinic during normal working hours:

*Because of my work. I used to leave my house early, early morning so I didn’t have enough time to go to the health centre every day. That’s why I left the treatment. Then, after my work, I used to arrive home late, when the health centre was closed. (026 - patient)*

## Clinical factors affecting outcomes

Clinical factors refer to aspects of patients’ treatment that affect their health and ability to finish their treatment schedule.

### Side-effects

All patients spoke of side-effects at some point during their treatment. A range of different side-effects was reported including loss of hearing and vision, nausea, loss of appetite, weakness, skin discolouration and low mood:

*When I take the pills, I think they are strong … And that causes me dizziness and I lose my vision … Right now, I can’t see you clearly … I have blurry vision. (004 - patient)*

Some patients explained that often the sight of the pills was enough to invoke nausea and vomiting:

*Now I am taking 18 pills every day … When I see the pills, I want to vomit. (010 - patient)*

Other patients reported the side-effects as being disabling, leaving them bed ridden for the remainder of their day.

*After the injection I have to stay in bed almost the whole day. (002 - patient)*

Ten patients and 8 HCPs spoke of strategies to minimise the severity of the side-effects. A common method was ensuring a meal prior to ingesting the pills:

*When they have side-effects sometimes they don’t want to continue, but when they come here, we explain to them that maybe they need to change the way they take the medications because maybe they could take them after having breakfast. (011 - HCP)*

Other patients found that spreading consumption of the pills over several hours helped:

*Yes, the nurses know about my problems and they help me … They let me take like 2 and then 2 again after a break. (010 - patient)*

## HCP-patient relationship affecting outcomes

The importance of a good, strong and an open HCP-patient relationship was highlighted by 13 patients and all 11 HCPs. The relationship can act as a foundation upon which good communication, patient confidence and trust can be established:

*The success of the treatment depends first on the treatment and then on the relationship you have with your patient. (011 - HCP)*

*The relationship between the doctor and the patient is very important. I think it is the foundation of the whole treatment and interaction. (017 - HCP)*

*With some of them I have a good relationship...but some of them look at you like you’re something weird. (013 - patient)*

### Communication

Good communication skills and empathy were cited by both groups as important skills for HCPs working with MDR-TB patients:

*We must try to be empathetic because we have to be in our patients’ shoes to know what it is like, what they are feeling and what they are thinking...We need to provide good treatment and support, so patients can feel comfortable enough to finish the treatment. (008 - HCP)*

In addition, HCPs understood that their role is to support the patient rather than establish a power-imbalance within the relationship:

*It’s not our role to say that “you have to do this” or “you have to do that”. (014 - HCP)*

Twelve patients and 10 HCPs described how good communication and making patients feel important and comfortable enough to share their worries or concerns about their treatment was vital:

*I try to give them attention… and try to make them feel important. I try to call them by their names and give them the chance to share things. I try to make them feel confident to share things with me and to follow the treatment. (011 - HCP)*

***Researcher*** *: Did you feel comfortable talking to the nurses about your concerns?*

*Yes, because I think they understood. (026 - patient)*

Many patients mentioned specific nurses whose encouragement had helped them during particularly difficult times:

*She [008 (HCP)] supported me when I was going to leave the treatment. I thought I was going to die and that’s when she supported me and got me back on the treatment. I am here thanks to her. (007 - patient)*

Furthermore, patients must visit the health centre every day for their observed treatment and therefore must feel welcome:

*I felt like the clinic was my home. (018 - patient)*

*We try to make the patient understand that they can come here any time they want and that they will have the support of all the staff. (012 - HCP)*

*If you don’t treat your patients well, they are not going to come back again. (020 - HCP)*

### Trust

Trust between HCPs and patients emerged as an important influencer of outcomes. Patients described trusting the advice of HCPs that was contradictory to the advice from their peers, while HCPs described trust that patients would follow their advice when they were not in the clinic:

***Researcher:*** *Does that [advice from peers] ever disagree with the advice from the doctors?*

*Yes, it contradicts it. Sometimes they disagree, but I believe, and I trust the doctors. (007 - patient)*

*We tell them that we need to follow the treatment schedule strictly. But we cannot be with the patient all the time, so we don’t know if they are taking natural medicines unless they tell us. (001 - HCP)*

### Transmission

Eight HCPs spoke of the danger of contracting MDR-TB themselves. The importance of self-care was evident not only to protect HCPs themselves, but also to protect patients:

*I try to make sure they understand why I am wearing the mask. I explain to them that they could transmit it to me and that’s why I am wearing it because if I get the illness, who is going to treat them? (014 - HCP)*

A protective mask, worn by most HCPs during consultations with patients, was mentioned several times as a necessary barrier to communication.

*It’s the only barrier. It’s the only barrier, but it’s necessary. (017 - HCP)*

While patients mainly spoke of the risk of transmission to their family, HCPs stressed the importance of reducing MDR-TB in the population to improve overall outcomes:

*The big problem is that patients sometimes know they have this illness, but don’t say anything or do anything and so keep on transmitting the illness to other people. So that’s the big problem here. (012 - HCP)*

*We make sure patients know about the ways they can pass it on to other people, so we can avoid people passing this illness on. In that way we are going to reduce the number of people who have MDR-TB. (021 - HCP)*

## Discussion

This study identified four principal themes that influence outcomes for MDR-TB: personal factors, external factors, clinical factors and the HCP-patient relationship. Moreover, separate analyses of the patient and HCP groups produced near identical thematic findings. This alignment of opinion has not previously been reported and adds support for a change in MDR-TB management strategies in Loreto, Peru.

### Personal factors

Patients and HCPs agreed that effective education enables the patient to come to terms with their diagnosis, deal with the side-effects of medication and take responsibility for their own health. The findings of this study also indicate that improved patient and population knowledge could facilitate engagement with treatment by encouraging belief in evidence-based medicine and dispelling health myths, belief in natural medicines and stigma. The importance of high standard patient knowledge and education is known [5, 20, 27]. However, this study offers deeper insight into the importance of the method of delivery. This includes how a variety of information sources could be used, most notably the internet and expert patients, and how information seeking behaviour should be encouraged.

Findings revealed a lack of effective patient and population level TB and MDR-TB education, which is associated with poor outcomes [43] and patient loss from treatment [26]. This study aligns with previous research showing that despite receiving pre-treatment information, being on treatment for a long time and receiving counselling throughout, many MDR-TB patients continue to have poor knowledge [20]. Thematic findings of this study indicate that improved population knowledge could reduce both the development of resistant strains and transmission of MDR-TB within the population.

Respondents emphasised the importance of a positive patient attitude towards MDR-TB treatment, which has previously been shown to impact patient adherence [20, 25, 27, 28, 44, 45] and MDR-TB outcomes [5, 46–49]. Patient motivation and attitude are considered difficult to modify since behavioural change is a complex and multifactorial process [23]. However, this study suggests that HCPs can encourage a positive attitude in patients through effective communication, empathy and by empowering the people around the patient to provide quality support.

Interviews provided a novel insight into the cultural importance of shamans and natural medicines in the Iquitos and Amazon region. We reported that most patients will see a shaman before seeing a doctor or nurse. HCPs indicated that a minority of shamans will refer patients to doctors, while others will attempt to cure MDR-TB with natural medicines alone, which are ineffective and delay presentation to health centres, and adversely affect patient outcomes [28, 50–54]. Belief in alternative medicines or negative perceptions of pharmaceutical treatment regimens has been associated with a detrimental impact on adherence [24, 55, 56]. However, this study has highlighted how deeply ingrained these beliefs are in the Loreto region and their impact on adherence and help-seeking behaviour.

The dangers of taking both natural and pharmaceutical medicines together were expressed by both patients and HCPs, which supports previous research [57]. Of interest, those patients who were near the end of their treatment regimen often advocated pharmaceutical medicines over natural medicines and would advise others starting their treatment to avoid natural medicines entirely. If expert patients with MDR-TB were encouraged to educate new patients it could reduce the negative impact of natural medicines on outcomes. The effectiveness of peer education has been confirmed in previous studies investigating other conditions [58, 59].

### External factors

The importance of a good support network around the patient to overcome the influence of structural factors and the socioeconomic impact of MDR-TB was identified.

In an area with high rates of poverty and no financial support available, patients often find themselves locked in a negative cycle due to the socioeconomic impact of MDR-TB on them and their families. Both groups in this study identified 2 options that the financial implications of MDR-TB commonly leave the patient with: firstly, to ignore medical advice and continue to work to earn enough money to provide for themselves and their family; secondly, to follow advice and rest, but risk not being able to provide. Patients who work are unable to rest or access health centres. However, patients who elect to rest must survive with less money, and struggle to pay for transport to access treatment and essential nutrition. Good nutrition is a vital part of MDR-TB prevention and treatment. For patients already living in poverty who cannot work, adequate nutrition is impossible, leading to longer treatment duration or treatment failure. The socioeconomic impact of TB and MDR-TB adversely influences adherence [5, 7, 60], ability to work [46, 61] and outcomes [21]. This study strongly emphasises the need for financial support for MDR-TB patients in Iquitos. Financial support in the form of food vouchers or direct income replacement has been proven to improve outcomes in other low-income settings in Nepal [25], South Africa [62] and elsewhere [25, 30–32]. A recent systematic review of all types of financial support for TB patients concluded that in low and middle income settings it was paramount to ensure that TB patients received appropriate income replacement to achieve optimal outcomes, with shorter treatment schedules making it cost effective [63]. A 2015 study by *Wingfield* et al. investigating the impact of incentivised cash transfers and counselling for TB patients from impoverished communities in Lima, Peru, found that patients responded positively to the socioeconomic intervention [64]. The findings of the study aided refinement of the program and it was hoped that they would provide a template for policy- and decision-makers in the Peruvian national government. This study reiterates the necessity of financial support for patients with MDR-TB. The framework proposed by *Wingfield* et al. should be used to guide implementation of a financial support program.

Patients and HCPs reported structural issues. A number of patients described difficulties accessing treatment, including the financial cost and distance to clinic, which is known to have a detrimental impact on outcomes for MDR-TB [15, 20, 21, 53, 65]. A resource poor health system, as in Iquitos, has been shown to adversely affect patient outcomes [15, 20, 24, 26, 66]. However, a proportion of HCPs argued that pragmatism and ingenuity allow them to continue to provide a satisfactory level of care despite a lack of sufficient resources. Both patients and HCPs described the value of a holistic approach to care including psychological and emotional support. Previous literature has described the importance of an individualised care strategy engaging and involving the patient [5, 23].

Patients and HCPs highlighted that issues relating to the deficit of personnel, funding and adequate and safe health centre facilities may be attenuated by the support network around the patient. Support and encouragement from HCPs, family and peers helps patients maintain a positive attitude towards their treatment, despite various challenges. Families invariably have to make sacrifices in order to support the ill family member and the impact is proportionally greater in a deprived environment. The vital support a family can provide includes emotional, psychological, nutritional and financial. HCP home visits positively influence outcome [5, 20]. However, this study has highlighted the important role of education of the family in empowering them to support the patient effectively and to prevent health myths and alternative medicine beliefs of the family from affecting the patient at home.

### Clinical factors

Side-effects are an unfortunate component of the patient journey through MDR-TB treatment and strategies to reduce them and help patients deal with them are essential. All patients described experiencing medication side-effects, which demonstrates the difficulties of taking highly toxic drugs. The way patients experience and perceive side-effects determines their ability to tolerate and continue with treatment. Patient experience of side-effects of MDR-TB treatment has previously been described [25, 28, 45] and is associated with poor adherence [20, 44].

### The HCP-patient relationship

The benefits of a strong, open and trusting relationship were highlighted by both patients and HCPs, as were the hazards of a poor relationship. The relationship acts as the basis for optimal outcomes through encouraging communication, patient confidence and trust. Although the importance of the HCP-patient relationship is recognised [20, 24, 67], no previous qualitative studies have carried out separate analyses to compare the two groups, which has provided a valuable insight into the dynamic of the relationship and shed light on ways in which a good relationship can be fostered. Good communication ensures that patients feel valued, confident and comfortable enough to share their worries with HCPs without fearing judgment. Adherence is essential to cure patients of MDR-TB, so it is important to ensure that they share any difficulties they are experiencing so that solutions can be found. In particular, empathy allows an HCP to imagine a patient’s situation and struggles while also giving the patient confidence that the HCP understands them. This study emphasised the importance of trust in the HCP-patient relationship, for example, patients must trust HCPs in order to believe in and follow their advice about the efficacy of pharmaceutical over natural medicines. Open dialogue and trust between patients and HCPs about negative perceptions of pharmaceutical medication has been shown to improve adherence and patient satisfaction for other conditions [68, 69]. The role of HCP-patient relationship in MDR-TB management must not be underestimated:

*The success of the treatment depends first on the treatment and then on the relationship you have with your patient. (011 -HCP)*

### Limitations

All interviews included a translator. One translator was used throughout the study to ensure consistency. Although translation accuracy checks post-pilot and post interviews found no obvious discrepancies, understanding and interpretation of the participant voice could have affected findings [70].

The setting of patient interviews varied based on convenience, and their availability. Where interviews occurred in health centres, it is possible that participants shared a narrative they thought TM, or an HCP would want to hear, e.g., fewer patient participants admitted taking natural medicines than was suggested by HCP participants. Variance of response suggests setting did not affect the data, but this is not guaranteed.

TM was neutral (not involved with the care of any patient) and prior to all interviews it was made clear that nothing the patient shared could affect their care. However, TM’s possible impact must be considered. Although measures were taken to reduce researcher bias, for example analytical triangulation with another independent researcher, TM’s central position in study design, data collection and analysis could have influenced findings. Results are considered relevant to other MDR-TB patients and their dedicated HCPs in Iquitos, as well as other resource poor settings in Latin America which hold similar cultural values. However, findings may not be reflected in dissimilar settings elsewhere.

Ethical approval did not allow for individual regimens to be published. However, Additional file 2 contains a table summary of the Peruvian national guidelines for the treatment of MDR-TB.

A comparison could have been made between patients with good adherence to their MDR-TB treatment and those who were poorly adherent. However, in Iquitos it became apparent that it would not be possible to contact MDR-TB patients who were currently poorly adherent because either they refused to participate, had died, or the risk of transmission was considered too great.

## Conclusions

This study has identified personal, external and clinical factors that influence MDR-TB outcomes as well as the importance of the HCP-patient relationship. Patient and general population knowledge could be improved with effective education programs using a variety of methods and information sources including the internet and expert patients. Improved knowledge, combined with other personal and external factors, could facilitate engagement with treatment by encouraging belief in evidence-based medicine and dispelling health myths, belief in natural medicines and stigma. HCPs should aim to work with shamans and communities to combat health myths and promote evidence-based medicine. An open, trusting and strong HCP-patient relationship can positively influence outcomes by establishing good communication, trust and a positive patient attitude. A positive attitude enables a patient to overcome adherence barriers and can also be encouraged by empowering the people around the patient to provide quality support. The socioeconomic impact of MDR-TB in a low-income setting is devastating not only to patients, but also their families. Financial support is necessary for the vast majority of patients and has been proven both effective and feasible in other low-income settings.

Although the eradication of MDR-TB is a considerable challenge, the findings of this study will contribute towards informing positive change towards this goal if they are considered and prioritised by local and national government. Future research should focus on increasing population-wide belief in evidence-based medicine and enhancing patient and population education.

## Additional files

Additional file 1: Summary of interview topic guide. Description: a table showing the important areas of conversation covered in every interview. (DOCX 13 kb)

Additional file 2: A table summarizing Peruvian national guidelines for the drugs available for the treatment of MDR-TB [13]. Description: A table showing all the available drugs for MDR-TB, the recommended daily dose, the maximum daily dose and the method of administration. (DOCX 15 kb)

## Data Availability

The datasets used and analysed during this study are available from the corresponding author on reasonable request.

## Abbreviations

HCP: Healthcare professional
MDR-TB: Multi-drug-resistant tuberculosis
TB: Tuberculosis
