# Supplementary material for: Improving outcomes for multi-drug-resistant tuberculosis in the Peruvian Amazon – a qualitative study exploring the experiences and perceptions of patients and healthcare professionals
Source: BMC Health Serv Res. 2019 Aug 22;19:594. doi: 10.1186/s12913-019-4429-y (PMC6704631; doi:10.1186/s12913-019-4429-y)
Supplement: Supplementary file 1 — Summary of interview topic guide. Description: a table showing the important areas of conversation covered in every interview. (DOCX 13 kb) [file 12913_2019_4429_MOESM1_ESM.docx]

**Supplementary File 1: Summary of interview topic guide**

| **Topic** | **Subtopics** |
| --- | --- |
| Personal factors influencing MDR-TB care | Patient knowledge and beliefs about TB and MDR-TB |
|  | Attitudes towards MDR-TB treatment and side-effects |
|  | Perceptions of future prospects and conceptualisation of illness |
| External factors influencing MDR-TB care for patients: | Patient perceptions of structural and health service factors (organisation) |
|  | Financial burden of MDR-TB |
|  | Other factors unanticipated by the researcher |
| The influence of a patients support network | Family, community and household influences on care |
|  | Patient experience of stigma |
|  | Media and information technology |
| Anything unanticipated by the researcher |  |
