# Supplementary material for: Improving outcomes for multi-drug-resistant tuberculosis in the Peruvian Amazon – a qualitative study exploring the experiences and perceptions of patients and healthcare professionals
Source: BMC Health Serv Res. 2019 Aug 22;19:594. doi: 10.1186/s12913-019-4429-y (PMC6704631; doi:10.1186/s12913-019-4429-y)
Supplement: Supplementary file 2 — A table summarizing Peruvian national guidelines for the drugs available for the treatment of MDR-TB [13]. Description: A table showing all the available drugs for MDR-TB, the recommended daily dose, the maximum daily dose and the method of administration. (DOCX 15 kb) [file 12913_2019_4429_MOESM2_ESM.docx]

**Supplementary File 2: A table summarizing Peruvian national guidelines for the drugs available for the treatment of MDR-TB [13]**

| **Drug** | **Daily dose** | **Maximum dose/day** | **Administration** |
| --- | --- | --- | --- |
| Para-amino salicylic acid | 150mg/kg | 12g | 4g sachet |
| Amikacin | 15mg/kg | 1g | 500 or 1000 mg ampule |
| Amoxicillin/clavulanic acid | 20-40mg/kg | 2000mg | 500/125mg tablet |
| Capreomycin | 15mg/kg | 1g | 1000mg ampule |
| Ciprofloxacin | 20-40mg/kg | 1500mg | 500mg tablet |
| Cycloserine | 15mg/kg | 1g | 250mg tablet |
| Clarithromycin | 25mg/kg | 1g | 500m tablet |
| Clofazimine | 15mg/kg | 200-300mg | 100mg tablet |
| Ethambutol | 20-25mg/kg | 1600mg | 400mg tablet |
| Streptomycin | 15mg/kg | 1g | 1000mg ampule |
| Ethionamide | 15mg/kg | 1g | 250mg tablet |
| Mipenem | 500-1000mg | 2000mg | 500mg ampule |
| Kanamycin | 15mg/kg | 1g | 1g ampule |
| Levofloxacin | 10-15mg/kg | 750-1000mg | 250/500mg tablet |
| Meropenemb | 20-40mg/kg | 3000mg | 500mg ampule |
| Moxifloxacin | 10mg/kg | 400mg | 400mg tablet |
| Pyrazinamide | 25-30mg/kg | 2000mg | 500mg tablet |
| Rifabutin | 5mg/kg | 300mg | 150mg tablet |
| Tioridazinabc | 0.5-3mg/kg | 200mg | 100mg tablet |
| Linezolidab | 10-20mg/kg | 600mg | 600mg tablet |
| Thioacetazoneab | 150mg | 150mg | 150mg tablet |
| a Not found in the National Single Request for Essential Medicines (Petitorio Nacional Único de Medicamentos Esenciales (PNUME))  b Used for the treatment of XDR-TB too  c Start with 25mg/day and increase progressively until maximum dose | | | |
